# Commentary: L-T4 Therapy in Enteric Malabsorptive Disorders

**DOI:** 10.3389/fendo.2021.696768

**Published:** 2021-06-25

**Authors:** Giuseppe Giuffrida, Alfredo Campennì, Salvatore Cannavò, Rosaria M. Ruggeri

**Affiliations:** ^1^ Department of Human Pathology of Adulthood and Childhood DETEV, University of Messina, Messina, Italy; ^2^ Nuclear Medicine Unit, Department of Biomedical Sciences and Morpho-Functional Imaging, University of Messina, Messina, Italy; ^3^ Endocrine Unit, “G. Martino” University Hospital, Messina, Italy; ^4^ Department of Clinical and Experimental Medicine, University of Messina, Messina, Italy

**Keywords:** hypothyroidism, levothyroxine therapy, malabsorption disorders, gastrointestinal diseases, cystic fibrosis

We really appreciated the paper recently published in Your Journal *Fallahi P, Ferrari SM, Elia G, Ragusa F, Paparo SR, Antonelli A. L-T4 Therapy in Enteric Malabsorptive Disorders. Front Endocrinol 2021;12:626371* (1) about the important clinical issue of levothyroxine (L-T4) malabsorption in patients affected by gastrointestinal disorders. The introduction of novel formulations, both the liquid ones and the soft gel capsules, has dramatically changed the outcomes of replacement therapy in these categories, allowing to achieve more adequate levels of TSH and better quality of life due to the reduced need for continuous dose changes.

The review by Fallahi and coworkers analyzed the impact of a wide spectrum of intestinal disorders on T4 treatment efficacy, in keeping with the interfering mechanisms and the pharmacologic features of T4 preparations. Herein we would like to point out, for the sake of completeness, that our research group has recently provided evidence that cystic fibrosis represents another cause of intestinal L-T4 malabsorption and that the novel formulations are efficacious in hypothyroid patients with CF to overcome malabsorption (2).

Cystic fibrosis (CF) is an autosomal recessive disease due to mutations of CF transmembrane conductance regulator (CFTR) gene on chromosome 7, which encodes an ion channel. When CFTR function is impaired, the consequent defect leads to an abnormal transport of chloride ions and bicarbonate in many organs and tissues. CF equally affects both sexes with a greatly variable prevalence depending on ethnicity, being estimated 1/1,800 to 1/5,000 in European Caucasians (3). Over the last few decades, the average lifetime and the life expectancy of CF patients has significantly increased, due to both an early diagnosis and specialized treatment in early stages of disease (4). Beside the peculiar lung involvement, CFTR mutations also interest pancreatic and biliary duct cells, causing defective bile flow and thick biliary secretion, pancreatic exocrine insufficiency, and nutrients malabsorption, finally leading to diabetes mellitus and biliary fibrosis (3). The above-mentioned elements can also contribute to drug malabsorption creating difficulties into reaching optimal therapeutical targets, like it is observed in hypothyroid patients in whom changing conditions and illnesses can determine the need to precisely adjust the doses of synthetic thyroid hormone.

We recently reported on the absorption profile of L-T4 in two patients affected by CF, a 44-year-old female, and a 39-year-old male, respectively, both in a state of post-surgical hypothyroidism (2). These subjects did not reach adequate TSH levels on weight-based doses of L-T4, so in order to analyze this finding we performed an absorption test by the administration of 600 ug of L-T4, measuring TSH, T4, and FT4 concentrations up to 4 h after drug ingestion. After LT4 load, T4 and FT4 remained below the lower reference limit in the first patient, while FT4 only slightly increased in the second one, confirming a true malabsorption in both subjects. The initiation of liquid LT4 formulation in both patients, at progressively lower daily doses (less than 2.0 ug/kg/d), led to target TSH ranges stable at the subsequent evaluations.

As demonstrated by Fallahi et al. and our experience, many factors can impair L-T4 absorption at gastrointestinal level, and in particular several diseases like *Helicobacter pylori* infection, celiac disease, inflammatory bowel diseases (IBDs), etc. In this context, CF must be mentioned since the mutations of CFTR gene correlate to pancreatic insufficiency, reduced biliary salt production, abnormal intestinal transit, and chronic intestinal inflammation, that ultimately concur to malabsorption in variable degrees, depending on disease severity. In particular, the increased L-T4 requirement associated with CF could be mainly due to defects in bile production and excretion, since the dysfunctional CFTR protein causes thickened secretions and biliary obstruction from plugging. This alteration not only leads to hepatocyte damage, with inflammation and fibrosis within the portal tracks, but can also contribute to suboptimal therapeutic regimens (5). Also, the lack of pancreatic enzymes and the consequent steatorrhea, as well as the abnormalities in intestinal transit, may result in increased fecal losses of L-T4, accounting for malabsorption (6). Moreover, some recent studies have also hypothesized a direct involvement of the gastro-intestinal tract probably due to a diffuse inflammation of the small bowel, as suggested by the finding of diffuse mucosal lesions and high levels of fecal calprotectin in some CF patients (7).

Prior to our report, there are only few data in the literature concerning this clinical issue. It is worth mentioning the experience described by Depasse et al. in the early ‘90s, a case of congenital hypothyroidism associated with CF, in which the two coexisting diseases complicated the management of thyroid dysfunction (6). In this male newborns the impaired pancreatic secretion and gastrointestinal transit abnormalities secondary to meconium ileus resulted in decreased absorption of L-T4, that was partially overcome by the administration of pancreatic enzymes, even if with greater doses than those required in newborns with congenital hypothyroidism to normalize TSH and FT4 levels (6). Recently, in our patients the use of the liquid L-T4 formulation with its improved pharmacokinetics has been demonstrated to bypass all the cited limitations, and permitted to reach target TSH levels that were missed by the conventional tablet formulation, as reported in other conditions (8). By shifting patients towards liquid L-T4 formulation we reached stable TSH levels, reducing the burden of frequent clinical and biochemical evaluations for the patients.

In summary, CF needs to be included in the list of enteric diseases involved in L-T4 impaired absorption, that should be always taken into account in these patients. L-T4 oral liquid formulation can overcome, at least in part, the reduced absorption of L-T4 in CF patients, leading to more tailored choices and a better management of hypothyroidism.

## Author Contributions

GG and RR conceived the commentary structure and performed the review of literature; RR, AC, and SC finally revised the manuscript. All authors contributed to the article and approved the submitted version.

## Conflict of Interest

The authors declare that the research was conducted in the absence of any commercial or financial relationships that could be construed as a potential conflict of interest.
